# Altered functional networks in long‐term unilateral hearing loss: A connectome analysis

**DOI:** 10.1002/brb3.912

**Published:** 2018-01-18

**Authors:** Yanyang Zhang, Zhiqi Mao, Shiyu Feng, Xinyun Liu, Lan Lan, Jun Zhang, Xinguang Yu

**Affiliations:** ^1^ Department of Neurosurgery PLA General Hospital Beijing China; ^2^ Department of Radiology PLA General Hospital Beijing China; ^3^ Department of Otolaryngology/Head and Neck Surgery PLA General Hospital Beijing China

**Keywords:** connectome, graph theory, resting‐state fMRI, unilateral hearing loss

## Abstract

**Introduction:**

In neuroimaging studies, long‐term unilateral hearing loss (UHL) is associated with functional changes in specific brain regions and connections; however, little is known regarding alterations in the topological organization of whole‐brain functional networks and whether these alterations are related to hearing behavior in UHL patients.

**Methods:**

We acquired resting‐state fMRI data from 21 patients with UHL caused by acoustic neuromas and 21 matched healthy controls. Whole‐brain functional networks were constructed by measuring interregional temporal correlations of 278 brain regions. Alterations in interregional functional connectivity and topological properties (e.g., small‐world, efficiency, and nodal centrality) were identified using graph‐theory analysis. The subjects also completed a battery of hearing behavior measures.

**Results:**

Both UHL patients and controls exhibited efficient small‐world properties in their functional networks. Compared with controls, UHL patients showed increased and decreased nodal centrality in distributed brain regions. Furthermore, the brain regions with significantly increased and decreased functional connections associated with UHL were components of the following important networks: (1) visual network; (2) higher‐order functional networks, including the default‐mode and attention networks; and (3) subcortical network and cerebellum. Intriguingly, the changes in intranetwork connections in UHL were significantly correlated with disease duration and hearing level.

**Conclusions:**

This study revealed connectome‐level alterations involved in multiple large‐scale networks related to sensory and higher‐level cognitive functions in long‐term UHL patients. These reorganizations of the brain in UHL patients may depend on the stage of deafness and hearing level. Together, our findings provided empirical evidence for understanding the neuroplastic mechanisms underlying hearing impairment, establishing potential biomarkers for monitoring the progression and further treatment effects for UHL patients.

## 1 INTRODUCTION

Characterized by asymmetric hearing input, unilateral hearing loss (UHL) poses many challenges, including difficulties in directional hearing and speech recognition (Kuppler, Lewis, & Evans, 2013; Lieu, Tye‐Murray, & Fu, 2012; Schmithorst, Plante, & Holland, 2014; Tibbetts et al., 2011). UHL is frequent in newborns (0.5/1,000 newborns), and the incidence increases with age (Kral, Hubka, Heid, & Tillein, 2013). Due to the relatively high incidence of UHL (Kuppler et al., 2013; Lieu et al., 2012; Schmithorst et al., 2014; Tibbetts et al., 2011) and the predominantly monaural therapy of congenital or acquired bilateral deafness with one cochlear implant (Gordon, Wong, & Papsin, 2013; Graham et al., 2009), the effects of UHL have attracted clinical interest (Kral, Heid, Hubka, & Tillein, 2013). Selecting a treatment for UHL requires weighing trade‐offs between intervention risks against expected benefits, which are currently difficult to predict. Intriguingly, the clinical outcome of cochlear implant treatment for UHL might be related to deafness‐induced cortical reorganization (Sandmann et al., 2012). Thus, under these circumstances, one aim of this study is to understand the underlying functional changes in the brain following long‐term UHL to provide potential biomarkers for identifying the disease and monitoring its progression.

Recent magnetic resonance imaging (MRI) studies mapped selected components of neural networks in UHL patients; the cumulative evidence of these studies suggested that hearing impairment is associated with network abnormalities. Using functional MRI, previous studies found that UHL not only altered the activity of sensory areas but also reshaped the regional and circuit functional organization of higher‐level networks, such as the default‐mode network (DMN) (Zhang et al., 2015), executive control network (Tibbetts et al., 2011), and language networks (Liu et al., 2015). By exploring the relationship between long‐term UHL and gray matter morphology, several structural imaging studies have demonstrated widespread involvement of various brain regions, including Heschl's gyrus, calcarine cortex, prefrontal cortex, and anterior cingulate cortex (Fan et al., 2015; Wang et al., 2016; Yang et al., 2014), hinting that structural changes may contribute to the formation of functionally abnormal brain networks in UHL patients. Although these previous imaging studies provided valuable information on neuro‐anatomical and functional changes in the brain in UHL from segregated brain areas, a whole‐system level (i.e., the connectome) understanding is still lacking.

Advances in the neuroimaging techniques of resting‐state functional MRI (rs‐fMRI) have enabled the mapping of high‐resolution functional networks through characterizing the patterns of inter‐region temporal correlations. From the connectomic perspective, the functions of the primary sensory cortex (e.g., auditory cortex), once thought to be pinnacles of modularity, are being redefined by recent evidence of cross‐modal interactions working together as large‐scale networks (Menon, 2011). Hence, deprivation of unilateral auditory input might compromise integral auditory perception, which could further propagate through widespread interregional connectivity to change the topological organization of intrinsic brain networks in UHL patients. Moreover, these topological changes could be quantitatively characterized using graph theoretical analysis (Bullmore & Sporns, 2009; Fornito, Zalesky, & Breakspear, 2013). More recently, one study suggested that UHL changed the topological organization of brain networks; however, this study focused only on patients with sudden UHL within the acute period (the disease duration is within 3 days; Xu et al., 2016). Thus, the topological organization of whole‐brain functional networks in long‐term UHL remains poorly understood.

In this study, we aimed to map functional connectomic changes present in long‐term UHL using rs‐fMRI. We employed an unbiased connectome‐wide approach to analyze group differences in every functional connection linking 278 regions distributed throughout the brain and changes in brain network organization using graph theoretical approaches. Our primary hypothesis is that the brain sensory regions typically affected by cross‐modal processing (e.g., auditory and visual processing regions) will differ between UHL patients and healthy controls (HCs). As an exploratory investigation, we also hypothesized possible differences in brain higher‐order networks, including the DMN and attention network, subserving the cognitive‐behavioral changes in UHL patients.

## 2 MATERIALS AND METHODS

### 2.1 Subjects

In this study, 21 long‐term UHL patients with primary ipsilateral acoustic neuroma (AN) were included. All patients had small tumors (diameter < 25 mm) with purely intrameatal or intraextrameatal tumor extension (classes T1–T2 according to the Hannover classification system; Matthies & Samii, 1997). Specifically, 12 patients had right‐sided UHL, and nine patients had left‐sided UHL. All UHL patients had postlingual deafness without tinnitus. Different from patients with sudden hearing loss (hearing loss duration < 3 days; Schreiber, Agrup, Haskard, & Luxon, 2010), all patients in this study had long‐term UHL (hearing loss duration > 2 months). We also selected 21 age‐ and sex‐matched HCs. All participants were right‐handed.

### 2.2 Hearing assessment

The hearing assessment was conducted in the Department of Otolaryngology/Head and Neck Surgery in PLA General Hospital. Participants were tested in a double‐walled, soundproof audiometric suite, which meets specifications for permissible ambient noise (ISO, 1994, 389‐3). We measured middle ear acoustic immittance for each ear to determine middle ear conditions, and no patients exhibited type B and C curves of tympanograms according to the Liden‐Jerger approach. Detailed descriptions of the middle ear acoustic immittance measurement can be found in the Data S1.

#### 2.2.1 Pure‐tone audiometry

**Figure 1:**
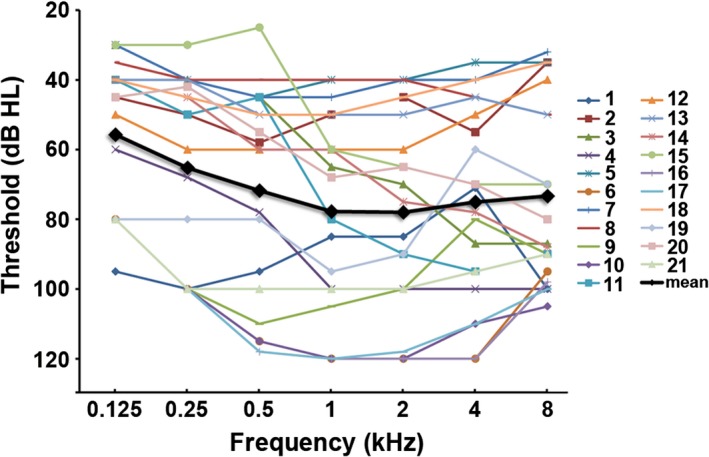
The frequency‐dependent hearing threshold of each subject in the affected ear

Pure‐tone audiometry was performed using a Madsen audiometer (Corena, Denmark) and standard TDH‐39 supra‐aural earphones (Grason‐Stadler, MN, USA). The audiological equipment was calibrated in accordance with ISO, 1989, 389‐1. A clinical audiologist collected pure‐tone hearing data under the standard Hughson‐Westlake procedure (steps: 10 dB down, 5 dB up; two of three). Audiometric thresholds for air‐conduction were obtained at the frequencies of 0.125, 0.25, 0.5, 1, 2, 4, and 8 kHz. Bone‐conduction thresholds were established at the frequencies of 0.25, 0.5, 1, 2, and 4 kHz. The procedures referred to GB/T 16403–1996. The audiogram of the affected ear for each patient is depicted in Figure 1.

The mean audiometric air‐conduction threshold over the frequencies of 0.5, 1, 2, and 4 kHz was calculated as the pure‐tone average (PTA) to reflect the hearing level (HL) for each ear. According to the World Health Organization (WHO) classification standard (1997), a PTA with 26~40 dB HL is defined as mild hearing impairment, 41~60 dB HL as moderate hearing impairment, 61~80 dB HL as severe hearing impairment, and >80 dB HL as profound hearing impairment. In this study, all patients were diagnosed with UHL with a hearing deficit in the affected ear (PTA value ≥ 40 dB HL) and normal hearing in the unaffected ear (PTA value ≤ 20 dB HL). The PTA values of control subjects were below 20 dB for both ears.

#### 2.2.2 Maximum phonetically balanced word recognition (PBmax)

PBmax was tested using a set of validated Mandarin monosyllable recognition test materials (Deng, Ji, & Yang, 2014). The test signals were delivered through custom‐made software to the external stimulation port of a calibrated Madsen audiometer (Corena, Denmark). The speech audiometry was in compliance with ISO, 2012, 8253‐3. Audio signals were presented monaurally to each participant's ear through a TDH‐39 earphone. Before the test began, the audiometer was adjusted with a calibration tone of 1 kHz, and the volume unit meter on the panel was returned to zero. At the very beginning of the test, several practice lists were presented to familiarize the subjects with both the speaker's voice and the test procedure. The subjects were instructed to listen carefully to a list of 50 monosyllables and iterate what they heard. The response of the subject to each test item was marked as true or false. PBmax for the test ear was identified with a signal level at 30 dB above the averaged thresholds of 0.5, 1, 2, and 4 kHz or the maximum comfortable level. PBmax was determined as the percentage of monosyllables correctly reported. The procedures referred to GB/T 17696‐1999. Generally, higher PBmax values indicate better speech recognition ability.

### 2.3 Image acquisition

Scans were performed using a GE750 3.0T scanner at PLA General Hospital. Participants were fitted with electrostatic headphone to mitigate acoustic scanner noise. The rs‐fMRI was obtained using an echo‐planar imaging (EPI) sequence with the following parameters: repetition time = 2,000 ms, echo time = 30 ms, flip angle = 90°, thickness/gap = 3.5 mm/0.5 mm, slices = 36, field of view = 224 × 224 mm^2^ and voxel size = 3.5 × 3.5 × 3.5 mm^3^, and a total of 240 volumes. The high‐resolution anatomical images were acquired using a sagittal fast spoiled gradient‐echo (FSPGR) T1‐weighted sequence with the following parameters: repetition time = 6.7 ms, echo time = 2.9 ms, flip angle = 7°, thickness = 1 mm, slices = 192, field of view = 256 × 256 mm^2^ and voxel size = 1 × 1 × 1 mm^3^. During the scan, all subjects were instructed to keep their eyes closed, to remain awake, and not to think of anything in particular. Data preprocessing was performed using Statistical Parametric Mapping (SPM8, http://www.fil.ion.ucl.ac.uk/spm) and Data Processing Assistant for Resting‐State fMRI (DPARSF; Chao‐Gan & Yu‐Feng, 2010), including removing first 10 time points, slice‐timing, realignment, spatial normalization, line detrending, nuisance covariates regression, and band‐pass filtering. For the details of preprocessing, see in Data S1.

### 2.4 Network construction

**Figure 2:**
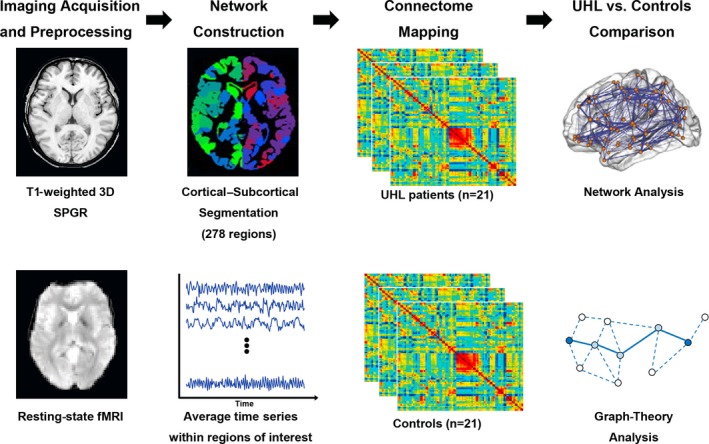
Analysis workflow. 3D, three‐dimensional; SPGR, spoiled gradient recalled; UHL, unilateral hearing loss

The analysis workflow is illustrated in Figure 2. To define the nodes for the brain network, the preprocessed rs‐fMRI data of each subject were divided into subregions using a template consisting of 278 nodes. This template was derived from a previous study that used functional connectivity data from 78 healthy individuals to form functionally homogenous brain regions (Shen, Tokoglu, Papademetris, & Constable, 2013). To define network edges, we computed the intrinsic functional connectivity for each pair of 278 nodes by computing Pearson correlation coefficients between the regional mean time series. This finding resulted in a symmetric connectivity matrix of size 278 × 278 for each subject. This method has been used in previous brain network studies (Chiang & Haneef, 2014; Pedersen, Omidvarnia, Walz, & Jackson, 2015; Zhang et al., 2011).

### 2.5 Network analyses

Here, we investigated the topological architecture of the functional connectome in UHL patients at three (global, nodal, and connectional) levels (Bullmore & Sporns, 2009). To avoid biases associated with using a single sparsity threshold, we applied a wide range of sparsity thresholds (5%–50% in steps of 5%), where prominent small‐world properties in brain networks were observed (Watts & Strogatz, 1998). At each of these thresholds, we calculated both global and node network metrics. For global network measures, we included the following: global efficiency (*E*_glob_), local efficiency (*E*loc), shortest path length (*Lp*), clustering coefficient (*Cp*), and small‐world parameters (λ, γ and σ; Rubinov & Sporns, 2010). For regional nodal characteristics, we considered the betweenness centrality (*BC*) metric similar to previous studies (Achard & Bullmore, 2007; Yasuda et al., 2015). *BC* is a widely used measure to identify the most central nodes in a graph (Bassett, Meyer‐Lindenberg, Achard, Duke, & Bullmore, 2006; Iturria‐Medina et al., 2011; Rubinov & Sporns, 2010). *BC* quantifies the number of short paths (between all other node pairs) that pass through a specific node divided by the total number of short paths in the entire network (Achard & Bullmore, 2007). Thus, *BC* can assess the degree of information flow of a brain region in whole networks, and a region with high *BC* indicates that it is part of “highly traveled paths” (Yasuda et al., 2015). We also calculated the regional efficiency to validate the results of nodal *BC*. Furthermore, for each network metric, we computed the area under the curve (AUC), which provides a summarized scalar for topological organization of brain networks independent of a single threshold selection. The integrated AUC metric has been used in previous brain network studies and is sensitive at detecting topological alterations in brain disorders (Lei et al., 2015; Zhang et al., 2011). At the connectional level, we identified regional pairs that showed between‐group differences in functional connections and further localized connected networks that showed significant changes in UHL patients.

### 2.6 Statistical analysis

We used nonparametric permutation tests to determine between‐group differences. Briefly, we initially calculated the between‐group differences in the mean of each global and regional network metric. We then randomly reallocated all of the values into two groups and recalculated the mean differences between the two randomized groups (10,000 permutations), thus an empirical distribution of the difference for each of graph‐based metric was obtained. The 95th percentiles of each distribution were used as critical values in a one‐tailed test to identify whether the observed between‐group differences were occurred by chance. Notably, before the permutation tests, effects of age and gender and education level were regressed by multiple linear regressions. Likewise, at connectional level, permutation tests were used to determine the significantly altered connectivity, and the false discovery rate (FDR) method with corrected *p *<* *.05 was used to correct for multiple comparisons (Genovese, Lazar, & Nichols, 2002).

To determine the relationship between brain network measures and clinical variables (hearing loss duration and hearing assessments), partial correlation analyses were conducted in the UHL group within the connections/nodes showing significant between‐group differences. The multiple comparisons were corrected with Bonferroni method.

The network analysis was performed using the GRETNA package (http://www.nitrc.org/projects/gretna/) and NBS package (http://www.nitrc.org/projects/nbs/). The results were visualized using the BrainNet Viewer package (http://www.nitrc.org/projects/bnv/).

## 3 RESULTS

### 3.1 Demographics and hearing assessment

There were no significant differences in age (*p *=* *.455), gender distribution (*p *=* *.525), and educational level (*p *=* *.973) between the UHL and HC groups. Compared with HCs, UHL patients had significantly higher PTA scores and lower PBmax scores in the affected ears (Table 1).

**Table 1:** Demographics and clinical data of all subjects

	UHL	Controls	*p* value
Age (years)	44.2 ± 3.5	42.8 ± 7.9	.455
Male/female	7/14	9/12	.525
Education (years)	11.1 ± 4.7	11.2 ± 4.3	.973
PTA of unaffected ear (dB HL)	17.6 ± 1.4	17.2 ± 1.2	.344
PTA of affected ear (dB HL)	73.3 ± 28.9	16.7 ± 2.1	<.001
PBmax of unaffected ear	0.99 ± 0.02	0.98 ± 0.02	.682
PBmax of affected ear	0.29 ± 0.33	0.99 ± 0.02	<.001
Duration of UHL (months)	34.0 ± 31.1	—	—

UHL, unilateral hearing loss; PTA, pure‐tone average; HL, hearing level; PBmax, maximum phonetically balanced word recognition score.

### 3.2 UHL‐related alterations in functional connections

**Figure 3:**
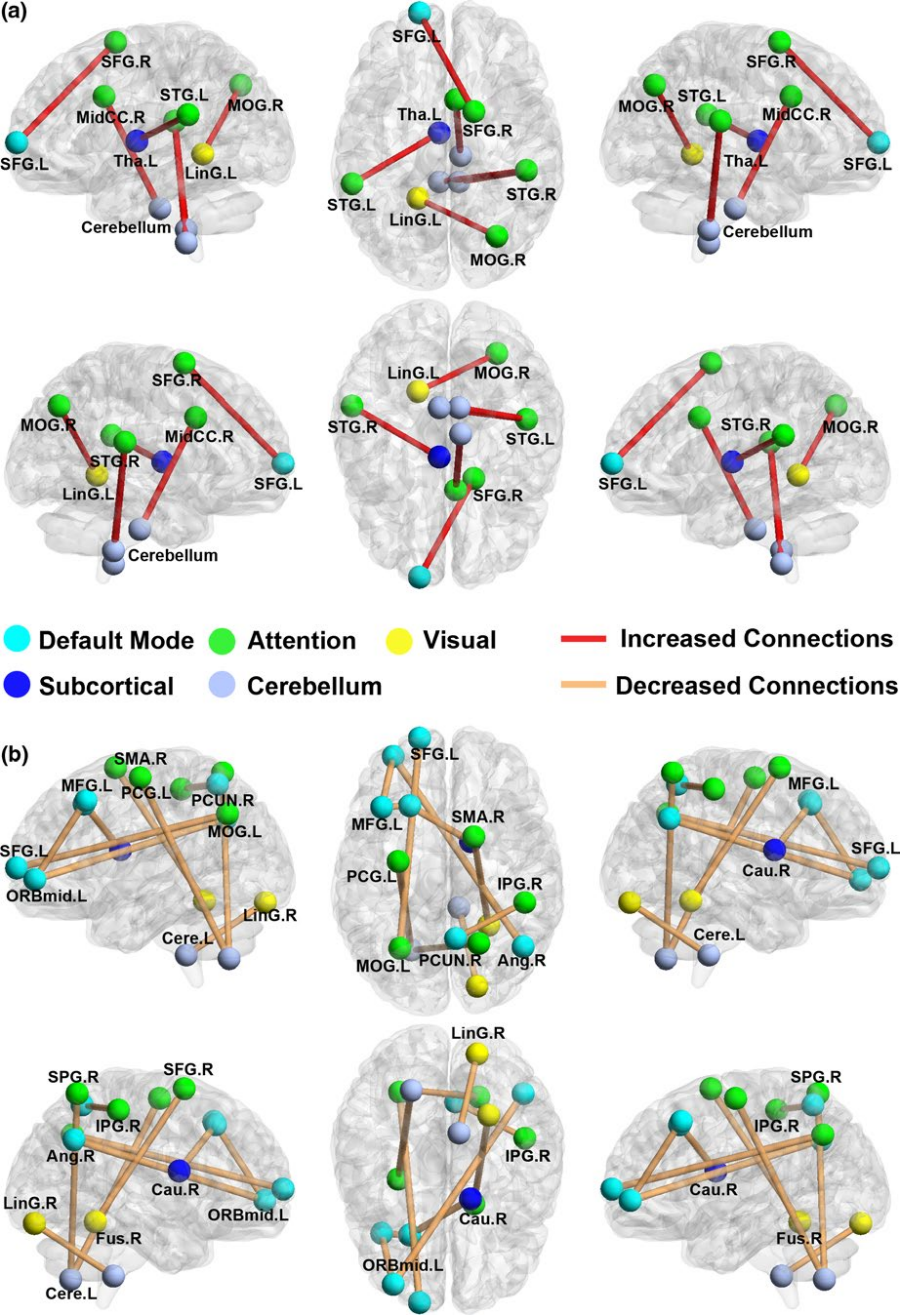
Region pairs exhibiting altered functional connections in the UHL group compared with the control group. (a) UHL patients relative to the control subjects showed significantly increased connectivity with 11 nodes and six connections (FDR corrected *p *<* *.05). (b) UHL patients relative to the control subjects showed significantly decreased connectivity with 16 nodes and nine edges (FDR corrected *p *<* *.05). The nodes and connections could be categorized into five functional networks: the attention network, default‐mode network, visual network, subcortical network, and cerebellum. The nodes and connections were mapped onto the cortical surfaces using the BrainNet Viewer package (www.nitrc.org/projects/bnv)

We identified significantly increased and decreased connections in the UHL patient group (*p *<* *.05, FDR‐corrected). The six increased connections involved 11 different nodes, including the bilateral superior frontal gyrus (SFG), right middle cingulate gyrus, bilateral superior temporal gyrus (STG), right middle occipital gyrus (MOG), left lingual gyrus, left thalamus, and cerebellum (Figure 3A). The nine decreased connections involved 16 different nodes, including the bilateral inferior parietal gyrus (IPG), right superior parietal gyrus (SPG), bilateral SFG, right sensory motor area (SMA), left MOG, orbital part of the left middle frontal gyrus (MFG), left MFG, right precuneus (PCUN), right angular gyrus, right fusiform gyrus, right lingual gyrus, right caudate nucleus, and left cerebellum (Figure 3B). These involved connections can be generally categorized into the following different functional networks: the attention network, DMN, visual network, subcortical network, and cerebellum (Figure 3).

### 3.3 UHL‐related alterations in regional nodal characteristics

**Figure 4:**
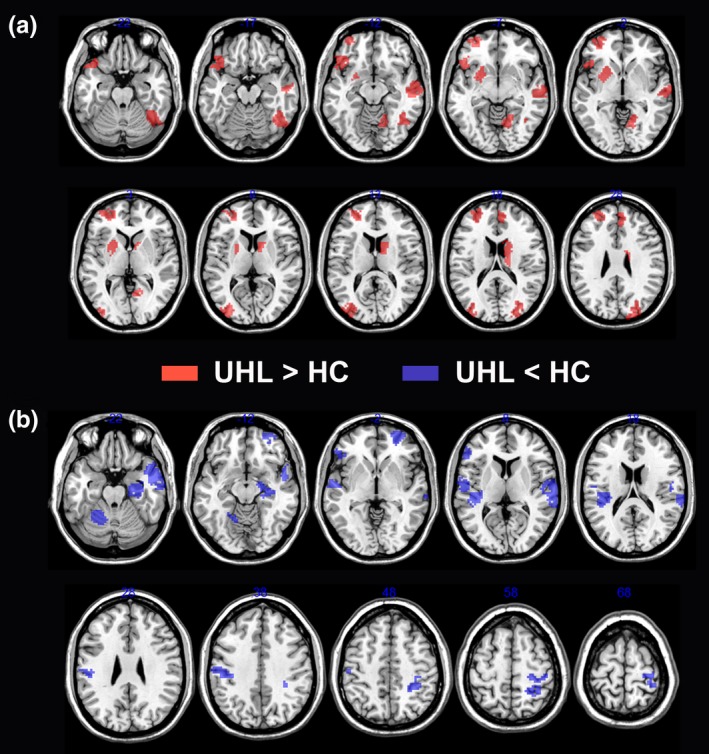
Brain regions showing altered nodal betweenness centrality in brain functional networks. (a) UHL patients relative to the control subjects showed significantly increased nodal betweenness centrality. These regions were predominantly located in visual network, default‐mode network, and subcortical network. (b) UHL patients relative to the control subjects showed significantly decreased nodal betweenness centrality. These regions were mainly located in auditory network, visual network, default‐mode network, and attention network. HC, healthy control; UHL, unilateral hearing loss

No regions survived after Bonferroni correction for multiple comparisons. As the subjects were relatively heterogeneous, and the sample size was small in each cohort, we chose to use an uncorrected threshold of 0.01 to balance providing control over false positives while maintaining sufficient power to detect differences. Compared with normal control subjects, UHL patients showed increased nodal betweenness in many brain regions, including the left putamen; right caudate nucleus; anterior cingulate cortex; several regions of the occipital (right lingual gyrus and bilateral MOG), frontal (orbital part of the left inferior frontal gyrus and left MFG), and temporal (right middle and inferior temporal gyrus) lobes; and right cerebellum (Figure 4A). Most of these regions were components of the visual network, DMN and subcortical network. Decreased nodal betweenness in UHL patients was predominantly located in the right hippocampal gyrus, bilateral Heschl's gyrus, bilateral superior and middle temporal gyrus, bilateral postcentral gyrus, the frontal (left inferior frontal gyrus and orbital part of the right MFG) lobe, and left cerebellum. These regions were mainly involved in the auditory network, visual network, DMN and attention network (Figure 4B). Moreover, the main results of BC were consistent with those of nodal efficiency (Figure S1).

### 3.4 Global network properties

**Figure 5:**
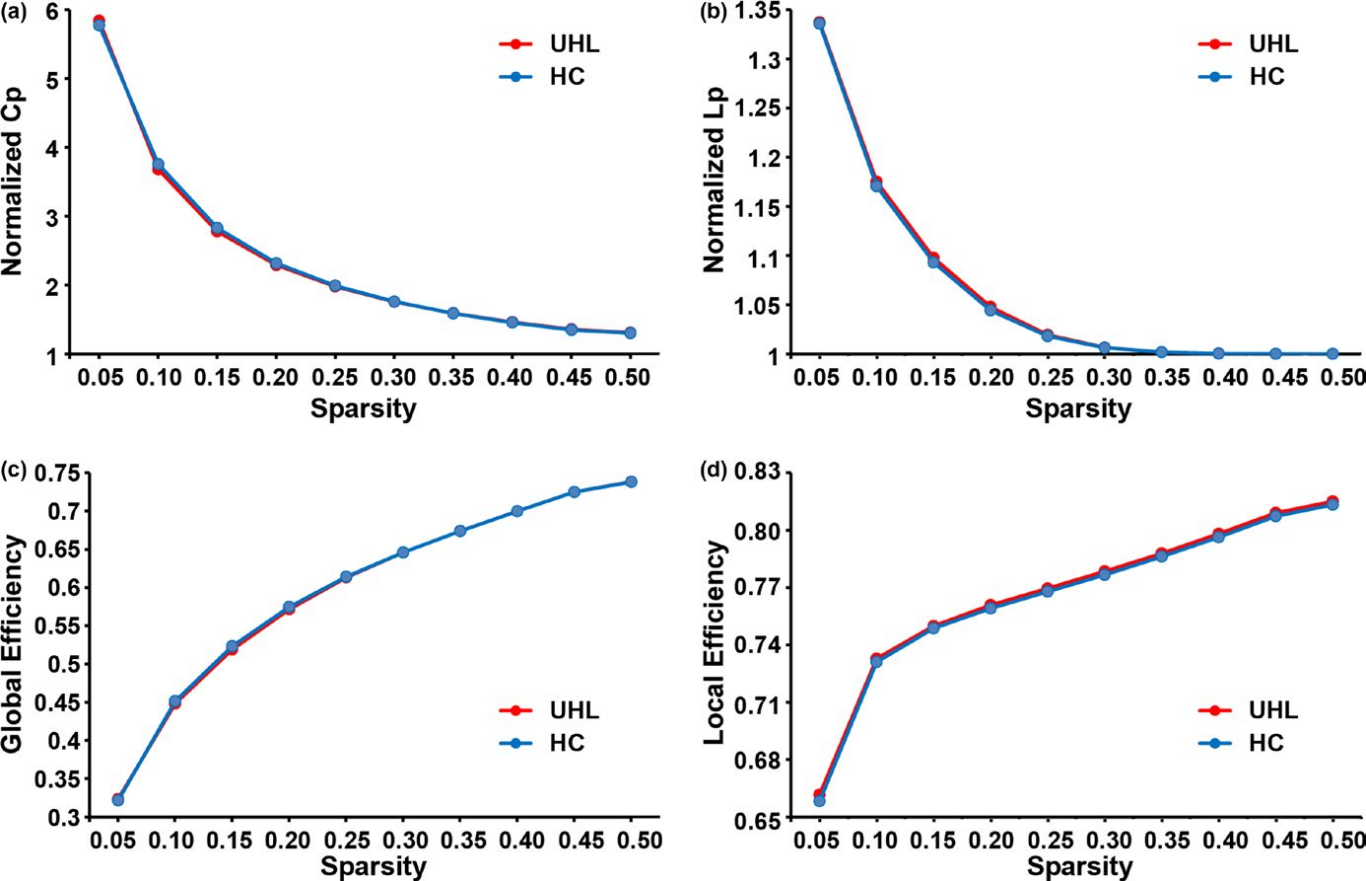
Both UHL patients and controls showed a small‐world organization of functional networks characterized by normalized clustering coefficients (*Cp*) > 1 (a) and normalized path lengths (*Lp*) ≈ 1 (b). The UHL patients and controls demonstrated almost equal global efficiency (c) and local efficiency (d) over the defined range of sparsity thresholds

There were no significant differences in any global parameter (*L*p, *C*p, *E*_glob_, *E*_loc_, λ, γ, and σ) of the whole‐brain functional networks between the two groups (Table 2). Both UHL patients and HCs exhibited efficient small‐world properties in the functional networks, characterized by almost identical path lengths (λ ≈ 1) but higher clustering coefficients (γ > 1) in the brain networks compared with those in matched random networks (Figure 5).

**Table 2:** Global network metrics in UHL patients and controls

Global network measures	UHL group (*n* = 21)	Control group (*n* = 21)	*t* value	*p* value
Cp	0.25 ± 0.014	0.25 ± 0.016	0.275	.785
Lp	0.78 ± 0.017	0.78 ± 0.018	0.134	.894
γ	1.02 ± 0.044	1.03 ± 0.039	−0.429	.670
λ	0.48 ± 0.007	0.48 ± 0.007	0.333	.741
σ	0.94 ± 0.046	0.95 ± 0.040	−0.502	.618
Global efficiency	0.27 ± 0.003	0.27 ± 0.004	−0.249	.804
Local efficiency	0.35 ± 0.006	0.35 ± 0.007	0.386	.702

Values are expressed as mean ± standard deviation.

### 3.5 Relationships between network measures and clinical performance

**Figure 6:**
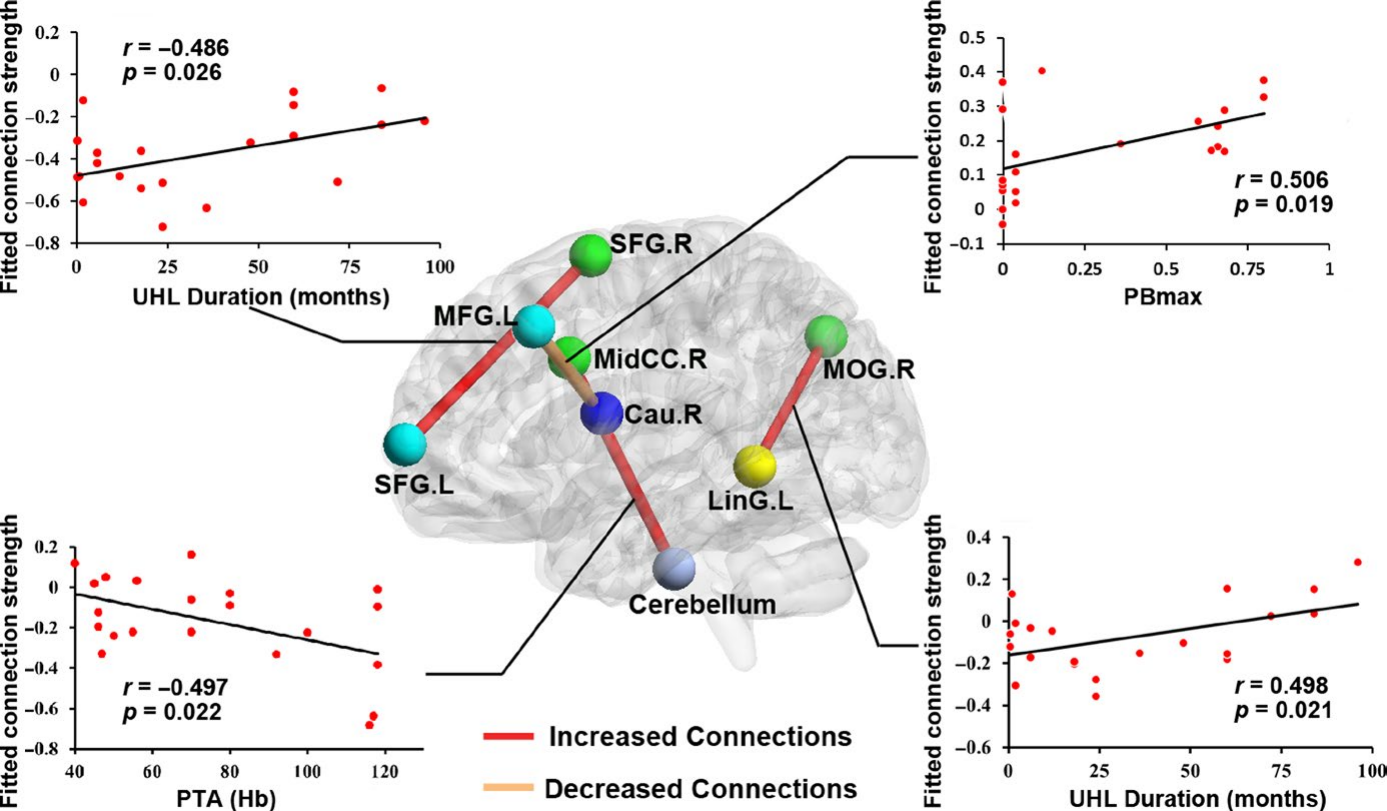
Relationships between network measures and clinical performance in UHL. The UHL duration was significantly correlated with the connections strength of the left SFG and right SFG, and the connections strength of left lingual gyrus and right MOG. We found a significant positive correlation between the connection strength of right middle cingulate gyrus and cerebellum with the PTA scores. We also found a significant positive correlation between the connection strength of left MFG and right caudate nucleus with the PBmax scores

Of note, correlations were not significant under the rigorous Bonferroni correction for multiple comparisons. Without correction, UHL duration was correlated with the connection strength of the left SFG and right SFG (*p *=* *.026) and the connection strength of the left lingual gyrus and right MOG (*p *=* *.021). PBmax scores were correlated with the connection strength of the left MFG and right caudate nucleus (*p *=* *.019). We also found a correlation between the connection strength of the right middle cingulate gyrus and cerebellum with PTA scores (*p *=* *.022, Figure 6).

## 4 DISCUSSION

To the best of our knowledge, the present study is the first demonstration of connectome‐level differences in functional networks between long‐term UHL patients and HCs. Although both groups exhibited efficient small‐world properties in their functional networks, UHL patients showed both altered connections and nodal centrality in the following networks: (1) visual network; (2) higher‐order functional networks including the attention network and DMN; and (3) subcortical network and cerebellum. The connectivity properties trended toward correlations with UHL‐related clinical variables and performance. Together, our study provides empirical evidence for changes in topological organization in functional networks in UHL patients.

Unilateral hearing loss patients and controls showed common small‐world properties of the functional networks. Thus, our findings indicate that UHL patients exhibited an optimized topological organization in brain networks despite the long‐term deprivation of unilateral auditory input. Specifically, the results were consistent with a recent rs‐fMRI study that found a typical small‐world network in sudden UHL patients (Xu et al., 2016). However, compared with control subjects, sudden UHL patients also showed a significantly increased clustering coefficient and a decreased characteristic path length in their brain networks, which was not detected in long‐term UHL patients. This discrepancy might reflect long‐term UHL‐related adaptive and compensatory functional reorganization to limit the consequences of neurological damage and help maintain topological organization (Hawellek, Hipp, Lewis, Corbetta, & Engel, 2011).

However, the absence of significant global topological differences does not preclude the existence of functional changes in nodes and connections in long‐term UHL patients. Indeed, the UHL‐related alterations in nodes and connections were found in the sensory network as well as in the higher‐order cognitive networks. Importantly, the sensory network involved was primarily located in the visual cortex. Consistently, previous structural imaging studies (Laugen, Brännström, Aarstad, Vassbotn, & Specht, 2016; Yang et al., 2014) have shown changes in gray matter volume in the lingual gyrus in patients with right‐sided hearing loss. Furthermore, using fMRI, Schmithorst et al. (2014) found significantly a different pattern of functional activation in visual processing regions while performing a classic receptive language test in UHL children. Wang et al. (2014) also found altered regional homogeneity (ReHo) in the bilateral calcarine cortices in UHL. In fact, anatomical studies in cats and nonhuman adult primates have demonstrated direct connections between auditory and visual cortical areas (Hall & Lomber, 2008; Rockland & Ojima, 2003). Thus, partial deprivation of one sensory modality (auditory) could affect the functions of the remaining intact sensory modalities (visual) (Schmithorst et al., 2014; Wang et al., 2014).

Previous neuroimaging studies have reported that long‐term UHL is associated with changes in the DMN (Wang et al., 2014; Zhang et al., 2015, 2017). This network underlies cognitive processes, such as emotional processing and self‐referential mental activity (Gusnard, Akbudak, Shulman, & Raichle, 2001), conflict monitoring (Kerns et al., 2004), memory retrieval (Wheeler et al., 2006) and cognitive control (Leech, Kamourieh, Beckmann, & Sharp, 2011). The altered connections in the DMN might consequently intrude on or disrupt ongoing cognitive performance, contributing to cognitive deficits in UHL patients, which is in line with clinical observations that UHL patients demonstrate significant difficulties with classroom behavior, cognition, and speech and language acquisition (Kuppler et al., 2013; Lieu et al., 2012; Schmithorst et al., 2014; Tibbetts et al., 2011). The attention network underlies attentional reorienting to salient relevant external stimuli or internal goals (Corbetta, Patel, & Shulman, 2008). Individuals with binaural hearing can produce relative intensity and phase differences in sound between both ears for spatial localization of a sound source. In fact, the ability of sound localization is critical for cognitive processing to improve self‐adaptation in a complex multisource environment (Ernst & Bülthoff, 2004). However, in UHL individuals, interaural differences are not available; thus, these individuals suffer from impaired sound localization ability (Wolter et al., 2016). We speculate that affected connections in the attention network underlie UHL‐related deficits in detecting sound localization in the environment. Moreover, specific altered connections exhibited a trend toward a correlation between illness duration and hearing assessment, raising the possibility that the reorganization of the brain in UHL patients may depend on the stage of deafness and HL. Together, our connectome‐level analysis suggested that the nodes and connections within higher‐order networks were disturbed in patients with long‐term UHL.

The subcortical network and cerebellum also exhibited altered functional connectivity in the UHL group. The cerebellum is anatomically and functionally connected with the frontal cortex through subcortical regions. Recent evidence indicates that the cerebellum has roles in cognitive processes in addition to motor function (Schmahmann & Caplan, 2006). Furthermore, neuroimaging studies have documented activation of the cerebellum during auditory, verbal, and language tasks (Bower & Parsons, 2003; Petacchi, Kaernbach, Ratnam, & Bower, 2011). A meta‐analysis found that the most active brain region responding to auditory‐related tasks in functional imaging, following the primary auditory cortex, was the cerebellum (Petacchi, Laird, Fox, & Bower, 2005). In the current study, we noted both increased and decreased functional connectivity between the cerebellum and other systems. In part, our results may reflect the substantial involvement of the cerebellum in psychoacoustic processing.

Neuroimaging studies, such as rs‐fMRI in this study, can detect putative brain network changes, which are difficult to observe through clinical measures alone (Cortese et al., 2012). Knowledge of the brain reorganization mechanisms associated with UHL might establish comprehensive clinical assessments and eventually elaborate personalized therapeutic intervention for UHL patients. Finally, such network‐based localization may facilitate tailored modulation of affected networks using techniques, such as noninvasive brain stimulation, with the therapeutic aim of alleviating clinical symptoms in UHL patients.

The present study had several limitations that merit additional investigation. First, functional brain networks constructed from the rs‐fMRI data were largely constrained by structural pathways (Honey et al., 2009). Thus, combining multimodal neuroimaging data could facilitate identification of structure–function relationships in UHL patients. Second, as a pilot rs‐fMRI study, although we characterized aberrant brain networks in UHL patients, whether these connectome‐level changes contributed to behavioral deficits were not systematically explored. Nevertheless, collective disruptions in intrinsic large‐scale networks were associated with parallel patterns of cognitive deficits in studies of many psychiatric and neurological disorders, such as depression, autism, schizophrenia, and dementia (Menon, 2011). Thus, we linked our current findings of brain network changes to behavioral deficits in UHL patients. Further works combining extensive psychoacoustic profiling and connectivity analysis are of great interest to uncover the neuronal bases underlying specific behavior and cognitive deficits in UHL. Finally, as the subjects were relatively heterogeneous, and the sample size was small in each cohort, the detection power was considerably reduced. Indeed, the results for nodal centrality and brain‐behavior correlations could not pass the stringent correction for multiple comparisons. Thus, these uncorrected findings should be considered only an exploratory analysis. Nonetheless, the reported results of altered nodal centrality within the sensory and higher‐order networks in UHL were highly consistent with those of connection‐level comparisons. Future studies are needed to increase the statistical power by selecting known regions of interest relevant to UHL or using a larger sample size.

## 5 CONCLUSIONS

Moving beyond models of hearing loss‐related changes focused on focal brain regions, the present exploratory study revealed connectome‐level alterations belonging to multiple large‐scale networks involved in sensory and higher‐level cognitive functions in long‐term UHL patients. Specific altered internetwork connections were significantly correlated with hearing performance in UHL. Our findings provided empirical evidence for understanding the neuroplastic mechanisms in UHL patients. Future studies might use functional neuroimaging assessments to formulate a comprehensive clinical treatment plan for UHL patients.

## References

[brb3912-bib-0001] Achard, S. , & Bullmore, E. (2007). Efficiency and cost of economical brain functional networks. PLoS Computational Biology, 3(2), e17 https://doi.org/10.1371/journal.pcbi.0030017 17274684 10.1371/journal.pcbi.0030017 PMC1794324

[brb3912-bib-0002] Bassett, D. S. , Meyer‐Lindenberg, A. , Achard, S. , Duke, T. , & Bullmore, E. (2006). Adaptive reconfiguration of fractal small‐world human brain functional networks. Proceedings of the National Academy of Sciences of the United States of America, 103, 19518–19523. https://doi.org/10.1073/pnas.0606005103 17159150 10.1073/pnas.0606005103 PMC1838565

[brb3912-bib-0003] Bower, J. M. , & Parsons, L. M. (2003). Rethinking the “lesser brain”. Scientific American, 289, 50–57. https://doi.org/10.1038/scientificamerican0803-48 10.1038/scientificamerican0803-48 12884538

[brb3912-bib-0004] Bullmore, E. , & Sporns, O. (2009). Complex brain networks: Graph theoretical analysis of structural and functional systems. Nature Reviews Neuroscience, 10(3), 186–198. https://doi.org/10.1038/nrn2575 19190637 10.1038/nrn2575

[brb3912-bib-0005] Chao‐Gan, Y. , & Yu‐Feng, Z. (2010). DPARSF: A MATLAB toolbox for “Pipeline” data analysis of resting‐state fMRI. Frontiers in Systems Neuroscience, 4, 13. 20577591 10.3389/fnsys.2010.00013 PMC2889691

[brb3912-bib-0006] Chiang, S. , & Haneef, Z. (2014). Graph theory findings in the pathophysiology of temporal lobe epilepsy. Clinical Neurophysiology, 125(7), 1295–1305. https://doi.org/10.1016/j.clinph.2014.04.004 24831083 10.1016/j.clinph.2014.04.004 PMC4281254

[brb3912-bib-0007] Corbetta, M. , Patel, G. , & Shulman, G. L. (2008). The reorienting system of the human brain: From environment to theory of mind. Neuron, 58, 306–324. https://doi.org/10.1016/j.neuron.2008.04.017 18466742 10.1016/j.neuron.2008.04.017 PMC2441869

[brb3912-bib-0100] Cortese, S. , Kelly, C. , Chabernaud, C. , Proal, E. , Di, M. A. , Milham, M. P. , & Castellanos, F. X. (2012). Toward systems neuroscience of ADHD: a meta‐analysis of 55 fMRI studies. The American journal of psychiatry, 169, 1038–1055. 22983386 10.1176/appi.ajp.2012.11101521 PMC3879048

[brb3912-bib-0008] Deng, X. S. , Ji, F. , & Yang, S. M. (2014). Correlation between maximum phonetically balanced word recognition score and pure‐tone auditory threshold in elder presbycusis patients over 80 years old. Acta Oto‐Laryngologica, 134, 168–172. https://doi.org/10.3109/00016489.2013.844855 24215216 10.3109/00016489.2013.844855

[brb3912-bib-0009] Ernst, M. O. , & Bülthoff, H. H. (2004). Merging the senses into a robust percept. Trends in Cognitive Sciences, 8, 162–169. https://doi.org/10.1016/j.tics.2004.02.002 15050512 10.1016/j.tics.2004.02.002

[brb3912-bib-0010] Fan, W. , Zhang, W. , Li, J. , Zhao, X. , Mella, G. , Lei, P. , … Xu, H. (2015). Altered contralateral auditory cortical morphology in unilateral sudden sensorineural hearing loss. Otology & Neurotology, 36(10), 1622–1627. https://doi.org/10.1097/MAO.0000000000000892 26595717 10.1097/MAO.0000000000000892 PMC4658668

[brb3912-bib-0011] Fornito, A. , Zalesky, A. , & Breakspear, M. (2013). Graph analysis of the human connectome: Promise, progress, and pitfalls. NeuroImage, 80, 426–444. https://doi.org/10.1016/j.neuroimage.2013.04.087 23643999 10.1016/j.neuroimage.2013.04.087

[brb3912-bib-0012] Genovese, C. R. , Lazar, N. A. , & Nichols, T. (2002). Thresholding of statistical maps in functional neuroimaging using the false discovery rate. NeuroImage, 15, 870–878. https://doi.org/10.1006/nimg.2001.1037 11906227 10.1006/nimg.2001.1037

[brb3912-bib-0013] Gordon, K. A. , Wong, D. D. , & Papsin, B. C. (2013). Bilateral input protects the cortex from unilaterally‐driven reorganization in children who are deaf. Brain, 136(Pt 5), 1609–1625. https://doi.org/10.1093/brain/awt052 23576127 10.1093/brain/awt052

[brb3912-bib-0014] Graham, J. , Vickers, D. , Eyles, J. , Brinton, J. , Al Malky, G. , Aleksy, W. , … Bray, M. (2009). Bilateral sequential cochlear implantation in the congenitally deaf child: Evidence to support the concept of a ‘critical age’ after which the second ear is less likely to provide an adequate level of speech perception on its own. Cochlear Implants International, 10(3), 119–141. https://doi.org/10.1179/cim.2009.10.3.119 19593746 10.1179/cim.2009.10.3.119

[brb3912-bib-0015] Gusnard, D. A. , Akbudak, E. , Shulman, G. L. , & Raichle, M. E. (2001). Medial prefrontal cortex and self‐referential mental activity: Relation to a default mode of brain function. Proceedings of the National Academy of Sciences of the United States of America, 98, 4259–4264. https://doi.org/10.1073/pnas.071043098 11259662 10.1073/pnas.071043098 PMC31213

[brb3912-bib-0016] Hall, A. J. , & Lomber, S. G. (2008). Auditory cortex projections target the peripheral field representation of primary visual cortex. Experimental Brain Research, 190, 413–430. https://doi.org/10.1007/s00221-008-1485-7 18641978 10.1007/s00221-008-1485-7

[brb3912-bib-0017] Hawellek, D. J. , Hipp, J. F. , Lewis, C. M. , Corbetta, M. , & Engel, A. K. (2011). Increased functional connectivity indicates the severity of cognitive impairment in multiple sclerosis. Proceedings of the National Academy of Sciences of the United States of America, 108, 19066–19071. https://doi.org/10.1073/pnas.1110024108 22065778 10.1073/pnas.1110024108 PMC3223469

[brb3912-bib-0018] Honey, C. J. , Sporns, O. , Cammoun, L. , Gigandet, X. , Thiran, J. P. , Meuli, R. , & Hagmann, P. (2009). Predicting human resting‐state functional connectivity from structural connectivity. Proceedings of the National Academy of Sciences of the United States of America, 106(6), 2035–2040. https://doi.org/10.1073/pnas.0811168106 19188601 10.1073/pnas.0811168106 PMC2634800

[brb3912-bib-0019] Iturria‐Medina, Y. , Pérez, F. A. , Morris, D. M. , Canales‐Rodríguez, E. J. , Haroon, H. A. , García, P. L. , … Melie‐García, L. (2011). Brain hemispheric structural efficiency and interconnectivity rightward asymmetry in human and nonhuman primates. Cerebral Cortex, 21, 56–67. https://doi.org/10.1093/cercor/bhq058 20382642 10.1093/cercor/bhq058

[brb3912-bib-0020] Kerns, J. G. , Cohen, J. D. , MacDonald, A. W. , Cho, R. Y. , Stenger, V. A. , & Carter, C. S. (2004). Anterior cingulate conflict monitoring and adjustments in control. Science, 303, 1023–1026. https://doi.org/10.1126/science.1089910 14963333 10.1126/science.1089910

[brb3912-bib-0021] Kral, A. , Heid, S. , Hubka, P. , & Tillein, J. (2013). Unilateral hearing during development: Hemispheric specificity in plastic reorganizations. Frontiers in Systems Neuroscience, 7, 93. 24348345 10.3389/fnsys.2013.00093 PMC3841817

[brb3912-bib-0022] Kral, A. , Hubka, P. , Heid, S. , & Tillein, J. (2013). Single‐sided deafness leads to unilateral aural preference within an early sensitive period. Brain: A Journal of Neurology, 136, 180–193. https://doi.org/10.1093/brain/aws305 23233722 10.1093/brain/aws305

[brb3912-bib-0023] Kuppler, K. , Lewis, M. , & Evans, A. K. (2013). A review of unilateral hearing loss and academic performance: Is it time to reassess traditional dogmata. International Journal of Pediatric Otorhinolaryngology, 77, 617–622. https://doi.org/10.1016/j.ijporl.2013.01.014 23474216 10.1016/j.ijporl.2013.01.014

[brb3912-bib-0024] Laugen, H. P. O. , Brännström, J. , Aarstad, H. J. , Vassbotn, F. S. , & Specht, K. (2016). Functional‐structural reorganisation of the neuronal network for auditory perception in subjects with unilateral hearing loss: Review of neuroimaging studies. Hearing Research, 332, 73–79. 26707432 10.1016/j.heares.2015.11.015

[brb3912-bib-0025] Leech, R. , Kamourieh, S. , Beckmann, C. F. , & Sharp, D. J. (2011). Fractionating the default mode network: Distinct contributions of the ventral and dorsal posterior cingulate cortex to cognitive control. Journal of Neuroscience, 31, 3217–3224. https://doi.org/10.1523/JNEUROSCI.5626-10.2011 21368033 10.1523/JNEUROSCI.5626-10.2011 PMC6623935

[brb3912-bib-0026] Lei, D. , Li, K. , Li, L. , Chen, F. , Huang, X. , Lui, S. , … Gong, Q. (2015). Disrupted functional brain connectome in patients with posttraumatic stress disorder. Radiology, 276(3), 818–827. https://doi.org/10.1148/radiol.15141700 25848901 10.1148/radiol.15141700

[brb3912-bib-0027] Lieu, J. E. , Tye‐Murray, N. , & Fu, Q. (2012). Longitudinal study of children with unilateral hearing loss. Laryngoscope, 122, 2088–2095. https://doi.org/10.1002/lary.23454 22865630 10.1002/lary.23454 PMC3467198

[brb3912-bib-0028] Liu, B. , Feng, Y. , Yang, M. , Chen, J. Y. , Li, J. , Huang, Z. C. , & Zhang, L. L. (2015). Functional connectivity in patients with sensorineural hearing loss using resting‐state MRI. American Journal of Audiology, 24, 145–152. https://doi.org/10.1044/2015_AJA-13-0068 25651853 10.1044/2015_AJA-13-0068

[brb3912-bib-0029] Matthies, C. , & Samii, M. (1997). Management of 1000 vestibular schwannomas (acoustic neuromas): Clinical presentation. Neurosurgery, 40(1), 1–9; discussion 9–10. 8971818 10.1097/00006123-199701000-00001

[brb3912-bib-0030] Menon, V. (2011). Large‐scale brain networks and psychopathology: A unifying triple network model. Trends in Cognitive Sciences, 15, 483–506. https://doi.org/10.1016/j.tics.2011.08.003 21908230 10.1016/j.tics.2011.08.003

[brb3912-bib-0031] Pedersen, M. , Omidvarnia, A. H. , Walz, J. M. , & Jackson, G. D. (2015). Increased segregation of brain networks in focal epilepsy: An fMRI graph theory finding. NeuroImage Clinical, 8, 536–542. https://doi.org/10.1016/j.nicl.2015.05.009 26110111 10.1016/j.nicl.2015.05.009 PMC4477107

[brb3912-bib-0032] Petacchi, A. , Kaernbach, C. , Ratnam, R. , & Bower, J. M. (2011). Increased activation of the human cerebellum during pitch discrimination: A positron emission tomography (PET) study. Hearing Research, 282, 35–48. https://doi.org/10.1016/j.heares.2011.09.008 22000998 10.1016/j.heares.2011.09.008

[brb3912-bib-0033] Petacchi, A. , Laird, A. R. , Fox, P. T. , & Bower, J. M. (2005). Cerebellum and auditory function: An ALE meta‐analysis of functional neuroimaging studies. Human Brain Mapping, 25, 118–128. https://doi.org/10.1002/(ISSN)1097-0193 15846816 10.1002/hbm.20137 PMC6871682

[brb3912-bib-0034] Rockland, K. S. , & Ojima, H. (2003). Multisensory convergence in calcarine visual areas in macaque monkey. International Journal of Psychophysiology, 50, 19–26. https://doi.org/10.1016/S0167-8760(03)00121-1 14511833 10.1016/s0167-8760(03)00121-1

[brb3912-bib-0035] Rubinov, M. , & Sporns, O. (2010). Complex network measures of brain connectivity: Uses and interpretations. NeuroImage, 52(3), 1059–1069. https://doi.org/10.1016/j.neuroimage.2009.10.003 19819337 10.1016/j.neuroimage.2009.10.003

[brb3912-bib-0036] Sandmann, P. , Dillier, N. , Eichele, T. , Meyer, M. , Kegel, A. , Pascual‐Marqui, R. D. , … Debener, S. (2012). Visual activation of auditory cortex reflects maladaptive plasticity in cochlear implant users. Brain, 135, 555–568. https://doi.org/10.1093/brain/awr329 22232592 10.1093/brain/awr329

[brb3912-bib-0037] Schmahmann, J. D. , & Caplan, D. (2006). Cognition, emotion and the cerebellum. Brain, 129, 290–292. https://doi.org/10.1093/brain/awh729 16434422 10.1093/brain/awh729

[brb3912-bib-0038] Schmithorst, V. J. , Plante, E. , & Holland, S. (2014). Unilateral deafness in children affects development of multi‐modal modulation and default mode networks. Frontiers in Human Neuroscience, 8, 164. 24723873 10.3389/fnhum.2014.00164 PMC3971169

[brb3912-bib-0039] Schreiber, B. E. , Agrup, C. , Haskard, D. O. , & Luxon, L. M. (2010). Sudden sensorineural hearing loss. Lancet, 375, 1203–1211. https://doi.org/10.1016/S0140-6736(09)62071-7 20362815 10.1016/S0140-6736(09)62071-7

[brb3912-bib-0040] Shen, X. , Tokoglu, F. , Papademetris, X. , & Constable, R. T. (2013). Groupwise whole‐brain parcellation from resting‐state fMRI data for network node identification. NeuroImage, 82, 403–415. https://doi.org/10.1016/j.neuroimage.2013.05.081 23747961 10.1016/j.neuroimage.2013.05.081 PMC3759540

[brb3912-bib-0041] Tibbetts, K. , Ead, B. , Umansky, A. , Coalson, R. , Schlaggar, B. L. , Firszt, J. B. , & Lieu, J. E. (2011). Interregional brain interactions in children with unilateral hearing loss. Otolaryngology ‐ Head and Neck Surgery, 144, 602–611. https://doi.org/10.1177/0194599810394954 21493243 10.1177/0194599810394954 PMC3433950

[brb3912-bib-0042] Wang, X. , Fan, Y. , Zhao, F. , Wang, Z. , Ge, J. , Zhang, K. , … Liu, P. (2014). Altered regional and circuit resting‐state activity associated with unilateral hearing loss. PLoS One, 9(5), e96126 https://doi.org/10.1371/journal.pone.0096126 24788317 10.1371/journal.pone.0096126 PMC4006821

[brb3912-bib-0043] Wang, X. , Xu, P. , Li, P. , Wang, Z. , Zhao, F. , Gao, Z. , … Liu, P. (2016). Alterations in gray matter volume due to unilateral hearing loss. Scientific Reports, 6, 25811 https://doi.org/10.1038/srep25811 27174521 10.1038/srep25811 PMC4865827

[brb3912-bib-0044] Watts, D. J. , & Strogatz, S. H. (1998). Collective dynamics of ‘small‐world’ networks. Nature, 393(6684), 440–442. https://doi.org/10.1038/30918 9623998 10.1038/30918

[brb3912-bib-0045] Wheeler, M. E. , Shulman, G. L. , Buckner, R. L. , Miezin, F. M. , Velanova, K. , & Petersen, S. E. (2006). Evidence for separate perceptual reactivation and search processes during remembering. Cerebral Cortex, 16, 949–959. https://doi.org/10.1093/cercor/bhj037 16162854 10.1093/cercor/bhj037

[brb3912-bib-0046] Wolter, N. E. , Cushing, S. L. , Vilchez‐Madrigal, L. D. , James, A. L. , Campos, J. , Papsin, B. C. , & Gordon, K. A. (2016). Unilateral hearing loss is associated with impaired balance in children: A pilot study. Otology & Neurotology, 37, 1589–1595. https://doi.org/10.1097/MAO.0000000000001218 27749751 10.1097/MAO.0000000000001218

[brb3912-bib-0047] Xu, H. , Fan, W. , Zhao, X. , Li, J. , Zhang, W. , Lei, P. , … Shi, H. (2016). Disrupted functional brain connectome in unilateral sudden sensorineural hearing loss. Hearing Research, 335, 138–148. https://doi.org/10.1016/j.heares.2016.02.016 26969260 10.1016/j.heares.2016.02.016

[brb3912-bib-0048] Yang, M. , Chen, H. J. , Liu, B. , Huang, Z. C. , Feng, Y. , Li, J. , … Teng, G. J. (2014). Brain structural and functional alterations in patients with unilateral hearing loss. Hearing Research, 316, 37–43. https://doi.org/10.1016/j.heares.2014.07.006 25093284 10.1016/j.heares.2014.07.006

[brb3912-bib-0049] Yasuda, C. L. , Chen, Z. , Beltramini, G. C. , Coan, A. C. , Morita, M. E. , Kubota, B. , … Gross, D. W. (2015). Aberrant topological patterns of brain structural network in temporal lobe epilepsy. Epilepsia, 56, 1992–2002. https://doi.org/10.1111/epi.13225 26530395 10.1111/epi.13225

[brb3912-bib-0050] Zhang, Y. , Mao, Z. , Feng, S. , Wang, W. , Zhang, J. , & Yu, X. (2017). Convergent and divergent functional connectivity patterns in patients with long‐term left‐sided and right‐sided deafness. Neuroscience Letters, 665, 74–79. 29175032 10.1016/j.neulet.2017.11.050

[brb3912-bib-0051] Zhang, J. , Wang, J. , Wu, Q. , Kuang, W. , Huang, X. , He, Y. , & Gong, Q. (2011). Disrupted brain connectivity networks in drug‐naive, first‐episode major depressive disorder. Biological Psychiatry, 70(4), 334–342. https://doi.org/10.1016/j.biopsych.2011.05.018 21791259 10.1016/j.biopsych.2011.05.018

[brb3912-bib-0052] Zhang, G. Y. , Yang, M. , Liu, B. , Huang, Z. C. , Chen, H. , Zhang, P. P. , … Teng, G. J. (2015). Changes in the default mode networks of individuals with long‐term unilateral sensorineural hearing loss. Neuroscience, 285, 333–342. https://doi.org/10.1016/j.neuroscience.2014.11.034 25463518 10.1016/j.neuroscience.2014.11.034

