# Supplementary figures and images for: Altered functional networks in long‐term unilateral hearing loss: A connectome analysis
Source: Brain Behav. 2018 Jan 18;8(2):e00912. doi: 10.1002/brb3.912 (PMC5822584; doi:10.1002/brb3.912)

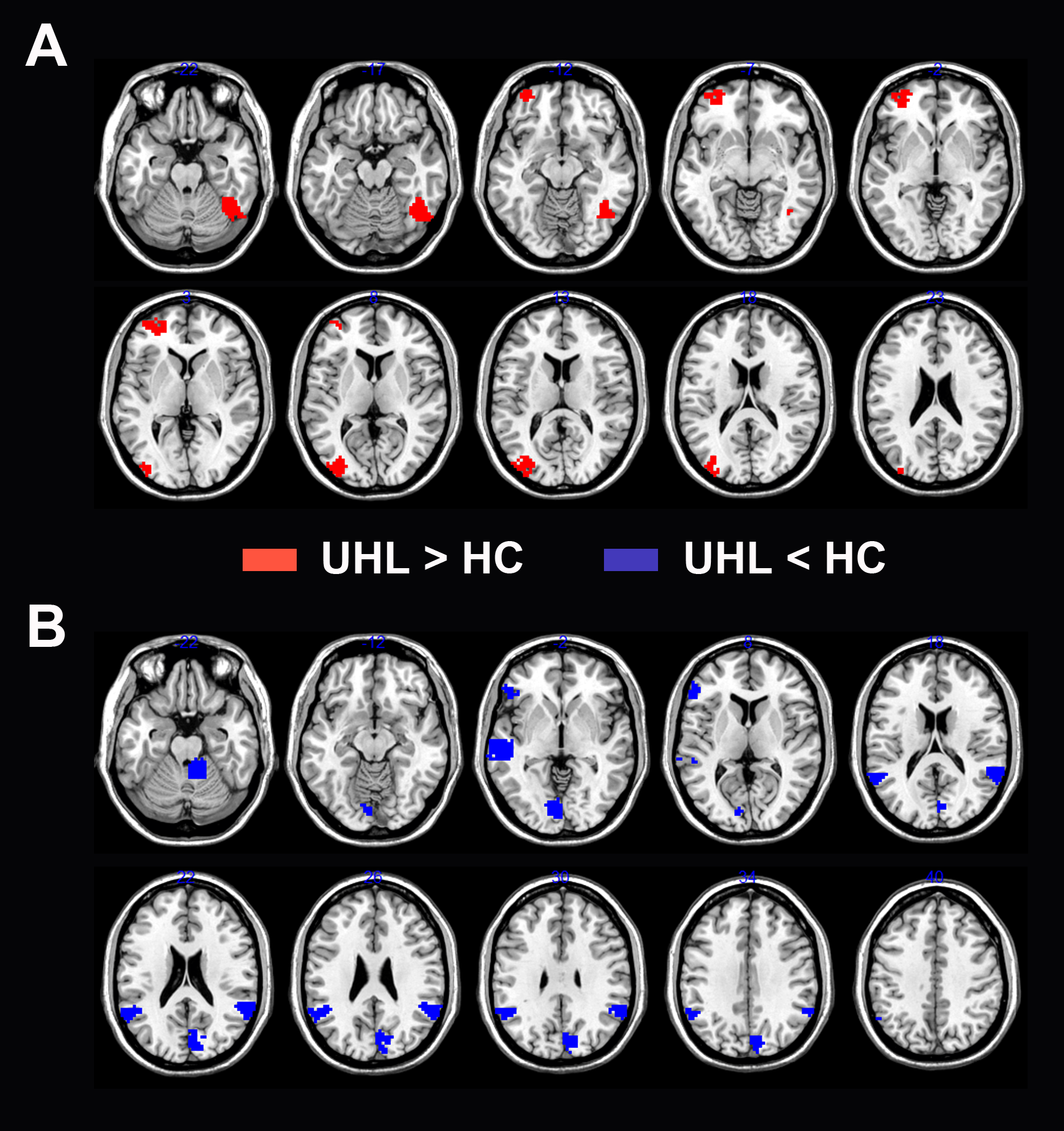

Supplement: Supplementary file 1 [file BRB3-8-e00912-s001.tif]
