# Supplementary material for: Altered functional networks in long‐term unilateral hearing loss: A connectome analysis
Source: Brain Behav. 2018 Jan 18;8(2):e00912. doi: 10.1002/brb3.912 (PMC5822584; doi:10.1002/brb3.912)
Supplement: Supplementary file 2 [file BRB3-8-e00912-s002.docx]

Measurement of middle ear acoustic immittance

We used Madsen OTOflex Middle Ear analyser (Denmark Madsen) to test tympanic pressure at -200 to 200 daPa, compliance and acoustic reflex on both ears. Tympanograms were drawn with pressure (mmH_2_O) and compliance (ml) as horizontal axis and vertical axis, respectively (Liu et al., 2015). Each tympanogram was typed automatically according to the following Liden-Jerger approach: (1) Type A, bell-shaped curve, 0-100 mmH_2_O for static compliance (peak), with a height of 0.3-1.4 ml, normal tympanogram; (2) Type As, tympanic pressure is normal, but the peak declines, <0.3 ml; (3) Type Ad, tympanic pressure is normal, but the peak increases, >1.4 ml; (4) Type B, flat curve without peak, indicating that tympanic compliance decreases due to tympanic diseases; (5) Type C, bell-shaped curve, but the height becomes normal, 0.3–0.4 ml, after the peak shifts till -100 mmH_2_O. None of ears exhibited type B and C curves in this study.

Data Preprocessing of fMRI

Data preprocessing was carried out using Statistical Parametric Mapping (SPM8, <http://www.fil.ion.ucl.ac.uk/spm>) and Data Processing Assistant for Resting-State fMRI (DPARSF) (Chao-Gan & Yu-Feng, 2010a). For scanner stabilization and participants’ adapting to the environment, the first ten volumes were discarded. The remaining functional scans were first corrected for within-scan acquisition time differences between slices and further realigned to the first volume to correct for inter-scan head motions. None of the participants were excluded based on the criterion of a displacement of more than 2 mm or an angular rotation of greater than 2 degrees in any direction. Next, the individual T1-weighted images were co-registered to the mean functional image after motion correction using a linear transformation and were then segmented into gray matter (GM), white matter, and cerebrospinal fluid by using a unified segmentation algorithm (Ashburner & Friston, 2005b). Further, the motion-corrected functional volumes were spatially normalized to the Montreal Neurological Institute (MNI) space using the transformation parameters estimated during unified segmentation and resampled to 3-mm isotropic voxels. The resulting normalized functional images were spatially smoothed (Gaussian kernel with a full width at half maximum of 4 mm) and were linearly detrended. Subsequently, the global signal, the white matter signal, the cerebrospinal fluid signal and 24 head motion parameters (6 motion parameters for the current the volume, 6 motion parameters for the previous volume and 12 corresponding squared items) were regressed out from the data. Finally, temporal band-pass filtering (0.01–0.08 Hz) was performed to reduce the effects of low-frequency drift and high-frequency physiological noise.

The definitions of these network properties are briefly described below, based on previous studies (Rubinov & Sporns, 2010c).

*Small-world properties.* In this study, we investigated the small-world properties (*Cp* and *Lp*) of the functional brain networks. The clustering coefficient of a node *i*, *C(i)*, which was defined as the likelihood of whether the neighborhoods were connected with each other or not. *Cp* was computed as the following:

$$Cp=\frac{1}{n}\sum_{i\in N} C(i)=\sum_{i\in N} \frac{\sum_{j,h\in N} a_{ij}a_{ih}a_{jh}}{K_{i}(K_{i}-1)}$$

where *K_i_* is the degree of node *i* and $a_{ij}$is the connection status between *i* and *j*. *Cp* of a network is the average of the clustering coefficient over all nodes and mirrors the extent of local interconnectivity or cliquishness in a network.

The path length between any pair of nodes (e.g., node *i* and node *j*) is defined as the sum of the edge lengths along this path. The shortest path length *L_i_*, is defined as the length of the path for node *i* and node *j* with the shortest length. The shortest path length of the network is computed as follows:

$$Lp=\frac{1}{n}\sum_{i\in N} L_{i}=\frac{1}{n}\sum_{i\in N} \frac{\sum_{j\in N,j\neq i}\text{ }L_{ij}}{n-1}$$

The *Lp* of a network quantifies the ability to propagate parallel information.

To further examine small-world properties, the *Cp* and *Lp* of brain networks were compared with those of random networks. In the present study, we generated 100 matched random networks, which had the same number of nodes, edges and degree distribution as the real networks (Maslov & Sneppen, 2002d). Furthermore, we computed the normalized shortest path length λ and normalized clustering coefficient γ using the following equation:

$$\gamma=\frac{C_{real}}{C_{random}}$$

$$\lambda=\frac{L_{real}}{L_{random}}$$

where $C_{random}$ and $L_{random}$ are the mean clustering coefficient and mean shortest path length of 100 matched random networks, respectively. A real network would be considered small-world if γ > 1 and λ ≈ 1(Watts & Strogatz, 1998e). In other words, a small-world network not only has a higher local interconnectivity but also has the shortest path length that is approximately equivalent to that of random networks. These two measurements can be summarized into a simple quantitative metric, small-worldness (σ = γ/λ), which is typically σ > 1 for small-world networks (Humphries, Gurney, & Prescott, 2007f).

*Network efficiency.* The global efficiency (*E*_glob_) measures the global efficiency of the parallel information transfer in the network (Latora & Marchiori, 2001g), which can be computed as follows:

where *L_ij_* is the shortest path length between node *i* and node *j*.

The local efficiency (*E*_loc_) reveals how much the network is fault tolerant, showing how efficient the communication is among the first neighbors of node *i* when it is removed. The local efficiency of a graph is computed as follows:

where *G_i_* denotes the subgraph composed of the nearest neighbors of node *i*.

*Regional nodal characteristics.* To determine regional nodal characteristics of the functional networks, we computed the betweenness centrality (*BC*) and nodal efficiency as follows:

$$BC\left(i\right)=\sum_{j\neq i\neq k\in N} \frac{\rho_{jk}\left(i\right)}{\rho_{jk}}$$

where $\rho_{jk}$ is the number of shortest paths between *j* and *k*, and $\rho_{jk}\left(i\right)$ is the number of shortest paths between *j* and *k* that pass through *i*. *BC* measures the relative importance of a node within the network. In turn, nodes that relay information in a range of network-wide nodes will have a high *BC*.

where is the shortest path length between node *i* and node *j* in *G*.

**REFERENCES**

Ashburner J, Friston KJ (2005b) Unified segmentation. Neuroimage 26:839-851.

Chao-Gan Y, Yu-Feng Z (2010a) DPARSF: A MATLAB Toolbox for "Pipeline" Data Analysis of Resting-State fMRI. Front Syst Neurosci 4:13.

Humphries, M.D., Gurney, K., Prescott, T.J., 2007f. Is there a brainstem substrate for action selection. Philos Trans R Soc Lond B Biol Sci. 362,1627-1639.

Latora, V., Marchiori, M., 2001g. Efficient behavior of small-world networks. Phys Rev Lett. 87,198701.

Liu, C. Q., Cheng, X. T., Zhu, Y. H., Shen, W. D., Bian, B. W., Cao, J. Y., ... (2015). Clinical observation on hearing conditions of centenarians in northern district of China. *Acta oto-laryngologica*, *135*, 451-458.

Maslov, S., Sneppen, K., 2002d. Specificity and stability in topology of protein networks. Science. 296,910-913.

Rubinov, M., Sporns, O., 2010c. Complex network measures of brain connectivity: uses and interpretations. Neuroimage. 52,1059-1069.

Watts, D.J., Strogatz, S.H., 1998e. Collective dynamics of 'small-world' networks. Nature. 393,440-442.
